# Food effect risk assessment in preformulation stage using material sparing μFLUX methodology^1^

**DOI:** 10.5599/admet.1476

**Published:** 2022-10-07

**Authors:** Corinne Jankovsky, Oksana Tsinman, Naveen K. Thakral

**Affiliations:** a Boehringer Ingelheim Pharmaceuticals, Inc., 900 Ridgebury Road, Ridgefield, Connecticut 06877, United States; b Pion Inc., 10 Cook St. Billerica, Massachusetts 01821, United States; # Presently at: Schrodinger, Inc., 1540 Broadway, New York, New York 10036, United States

**Keywords:** Food effect, bioavailability, solubility, biorelevant medium, predictive dissolution, permeability, flux, diffusion, clinical trial

## Abstract

The intake of food and meal type can strongly impact the bioavailability of orally administered drugs and can consequently impact drug efficacy and safety. During the early stages of drug development, only a small amount of drug substance is available, and the solubility difference between fasted state simulated intestinal fluid and fed state simulated intestinal fluid may provide an early indication about the probable food effect. But higher drug solubility in fed state simulated intestinal fluid may not always results in an increased oral absorption. In the present research, we demonstrated using 11 model compounds that in addition to the drug dissolution in biorelevant media, the evaluation of the diffusion flux of a drug in solution, across artificial lipid coated membrane, where only the unbound drug crosses the membrane, is a reliable way to predict the food effect. Although, the combination of dissolution and diffusion flux may not reliably predict the food effect in case of drugs undergoing intestinal metabolism or when transporters are involved in the drug absorption, the technique generally provides good information about the food effect at very early stages of drug development that may help in designing a clinical plan by adjusting the drug dose in the fed state.

## Introduction

Oral route of drug administration is convenient and preferred for patient centric drug development [1]. But there is high drug pharmacokinetics (PK) variability associated with the oral route. The oral drug absorption may be influenced by many intrinsic and extrinsic factors, thereby inducing the variability in systemic drug exposure. Food is one of the prime extrinsic factors that influence the oral drug absorption. Food intake alters the gastrointestinal environment, for instance, stomach pH, chyme viscosity, bile concentration and luminal fluid volume. Food intake is also associated with the physiological and sensory responses such as prolonged gastric emptying time, increased splanchnic blood flow, and change in metabolizing enzyme activity, in addition to the altered hydrodynamics. Food components may interact with drugs to form complex and can also influence their physicochemical properties like dissolution rate, and ionization state [2,3]. This complex interplay of altered gastrointestinal milieu, drug properties and physiological responses may impact the drug absorption in the fed state. The food may influence the drug absorption in three ways – reduced and delayed (negative food effect) [4], increased and accelerated (positive food effect) [5], or no food effect [6]. Following food intake, bile salts and acid are released into the gut and emulsify dietary fat for digestion [7]. The average bile salts concentration in 20 human volunteers was found to be 4.61 mM (fasting) and 12.65 mM (fed) [8]. Higher concentration of bile salts and acid in fed state helps in the solubilization of the poorly water-soluble drugs through micelle formation [3]. But solubilization of drugs may not always increase their oral absorption. The bile micelles may “entrap” and reduce the free drug concentration available at the epithelial membrane surface. Micelle formation may also reduce the diffusion coefficient of the drug in the unstirred water layer adjacent to the epithelial membrane and may lead to a decrease in effective permeability. Kiyohiko Sugano reviewed the effect of bile micelles on solubility, dissolution rate and permeability, and thereby overall food effect on the oral drug absorption [9,10]. High fat contents of food may also result in an inhibitory effect on intestinal efflux transporters and hence increase in the bioavailability of drugs that are substrate of these efflux transporters. On similar lines inhibition of uptake transporters by fat contents may lead to decrease in bioavailability of drugs reliant of these transporters for the absorption [11]. In several cases negative food effect has been observed for the drugs that are substrate to the efflux transporters. It has been reported that the prolonged gastric emptying time in fed state leads to decreased drug concentration in the intestinal lumen to saturate the efflux transporters, and therefore increase the drug transport efficiency in fed state as compared to the fasted state [3].

The change in PK induced by co-administration of food can have serious repercussion for narrow therapeutic-index drugs. A positive food effect may compromise safety if systemic exposure exceeds maximum tolerable dose. Similarly, a negative food effect may compromise efficacy of the drugs by decreasing the systemic absorption [5]. Therefore, assessing the effect of food on the absorption of a drug is critical to optimize the safety and efficacy of the final drug product. The assessment is also important for the accurate instructions to be provided on the product label for drug administration in relation to food. The United States Food and Drug Administration (FDA) has provided recommendations in the form of guidance document to conduct food-effect studies for orally administered drug products as part of investigational new drug applications (INDs), new drug applications (NDAs), and supplements to these applications. The document recommends the assessments of the food effect (with a high fat meal) on a new drug during phase 1 clinical trials (as part of the first-in-human trials) to decide if a drug should be administered with food in the trials until a final commercial formulation is identified. For the data analyses the guidance document recommends evaluating the effect of food on PK parameters, including total exposure of the drug (area under the curve; AUC_0-∞_, AUC_0-t_), the peak concentration of the drug and time to peak (*C*_max_ and *T*_max_), terminal elimination half-life (*t*_1/2_), the apparent clearance, the apparent volume of distribution, and lag-time in achieving *T*_max_ (*T*_lag_). Based on log transformed data, when the 90 percent confidence interval for the ratio of the population geometric means between fed and fasted treatments fall outside of 80–125% for AUC (AUC_0-∞_ or AUC_0-t_ when appropriate) and *C*_max_, the FDA considers the presence of food effect. The clinical significance of any difference in *T*_max_ and *T*_lag_, if any, should also be considered [12].

Due to the complex nature of food effect, pharmaceutical industry follows an integrated approach for the assessment during early phase of discovery and development. A combination of studies in preclinical species, predictive *in vitro* models and physiologically based absorption modeling are utilized before first-in-human clinical trials. FDA has provided recommendations for the development of clinically relevant dissolution specifications (method and acceptance criteria), and for the development, evaluation, and use of physiologically based pharmacokinetic (PBPK) analyses for biopharmaceutics applications in support of drug product development [13]. Various *in silico*, *in vitro*, and *in vivo* tools and techniques have been reviewed for the food effect prediction during drug development [2,14]. Characterization of the luminal environment after food intake, selection of appropriate biorelevant dissolution media for fed state and various *in vitro* methodologies for evaluating drug product performance in the fed state has also been reviewed [15]. Practically, during the early stages of drug development only a few milligrams of drug substance (DS) is available, not sufficient even for the pre-clinical formulation development. This does not leave a lot of room to improve wettability and surface area limitations of "grease ball molecules" at this stage of development. The commonly used techniques to predict the food effect [2,14] require either large quantities of DS or drug product (DP) formulation for the test. This warrants for a technique to reliably predict the food effect using a small quantity of DS and provides a middle ground between physicochemical throughput screening and a final product testing. The solubility difference between fasted state simulated intestinal fluid (FaSSIF) and fed state simulated intestinal fluid (FeSSIF), may provide an initial indication about the probable food effect, but as mentioned above, an increase in drug solubility in FeSSIF medium may not always results in an increase in oral absorption. Therefore, food effect prediction based on solubility in biorelevant media may be misleading. In the fed state, the micelles and colloidal species formed by bile salts and lecithin may help in solubilization of insoluble drugs. The drug molecules may form a complex with bile salts or might get “entrapped” into the micellar core. As only the free drug molecules in solution (unbound form) of the drug is available for the absorption through the epithelial membrane, the equilibrium solubility data and the total drug dissolved may not reliably predict the food effect. In the present research, we hypothesized that in addition to the drug dissolution in biorelevant media, the evaluation of the diffusion flux (membrane transport rate across artificial, lipid coated membrane, using side-by-side diffusion cells) of a drug in solution, where only the unbound drug crosses the membrane, is more reliable way to predict the food effect (FE). To test the hypothesis, we utilized eleven model compounds belonging to biopharmaceutics classification system (BCS) I - IV categories.

## Material and methods

### Materials

Amiodarone hydrochloride, celecoxib, clopidogrel bisulfate, danazol, fluoxetine hydrochloride, furosemide, nefazodone hydrochloride, nifedipine, zidovudine and isoniazid were purchased from Sigma Aldrich Inc. (Milwaukee, WI, USA). Griseofulvin was provided by Pion. The gastrointestinal tract lipid (GIT-0 PN 110669), the acceptor sink-buffer (ASB PN 110139) and the PVDF (polyvinylidene difluoride) filter support (PN 120875) were purchased from Pion Inc. (Billerica, MA, USA). FaSSIF buffer concentrate, FeSSIF buffer concentrate and the simulated intestinal powder version I FaSSIF/FeSSIF/FaSSGF, were purchased from Biorelevant.com (London U.K.). DMSO (dimethyl sulfoxide) and methanol (HPLC Plus grade) used to prepare the stock solution for standards were purchased from Sigma Aldrich (Milwaukee, WI, USA).

Media preparation

Dissolution/flux tests were performed using the FaSSIF, FeSSIF version 1 to simulate the fasted state and fed state intestinal fluids (Table-1 for media composition). FaSSIF and FeSSIF were prepared using the buffer concentrate method with FaSGF/FaSSIF/FeSSIF powder as described in Biorelevant Media preparation tool on their website (Biorelevant.com). Table-1 summarizes the composition of the intestinal media used in this study.

### Methods

Dissolution/flux tests were performed using a μFLUX apparatus (Pion Inc., Billerica, MA, USA) with 4 side-by-side donor-receiver chambers separated by a lipophilic barrier. Prior to the assay, the artificial GIT-mimicking membrane was prepared by impregnating a PVDF filter support material (polyvinylidenfluoride, 1.54 cm^2^ open area, 0.45 μm pore size, 120 μm thickness, 70 % nominal porosity) with 25 μL of 20 % lecithin in dodecane lipid solution (GIT Lipid, Pion Inc., Billerica, MA, USA). The donor chambers were then filled with 20 ml of biorelevant media (FaSSIF or FeSSIF) while the receiver compartments were filled with 20 ml of acceptor sink buffer (ASB, Pion Inc., Billerica, MA, USA). The ASB is a HEPES (4-(2-hydroxyethyl)-1-piperazineethanesulfonic acid) based pH 7.4 buffer containing chemical scavengers (surfactants micelles) that allow to maintain sink conditions during the experiments. The composition of the membrane and the receiver solution are identical to the Double-Sink PAMPA model [17], a non-cell-based method predicting of passive transcellular intestinal absorption. The temperature in both chambers was maintained at 37 °C and the media were stirred at 100 rpm.

Model compounds were added at the start of the experiment in the donor chambers as unformulated powder at a solid load equivalent to the lowest marketed dose normalized to 250 mL stomach volume. In some cases, to counter for experimental limitation due to low signal detection or high turbidity, a higher or lower dose was selected. The dose used for each model compound can be found in Table 3. The UV spectra in donor and receiver chambers were monitored simultaneously using UV-vis fiber optic probes (tips path length 2-20 mm) connected to the Rainbow® instrument (Pion Inc., Billerica, MA, USA) and were analyzed using AuPro software (revision 6 or higher) to determine the apparent drug concentration in both chambers.

Standards for each model compound were prepared by sequential addition of a stock solution of pure API pre-dissolved in organic solvents (either methanol or DMSO) to the corresponding medium. The area under the 2^nd^ derivative spectra curves was used to calculate the concentrations. The wavelength range was selected individually for each compound in the way to avoid sensitivity issues. Linearity of the standard curves in the selected wavelength regions were characterized by r^2^ ≥ 0.998. The drug concentration in the donor and receiver chambers was calculated from the UV absorbance of the samples using the corresponding standard curve. For samples with low permeability, an additional μFLUX donor-receiver pair containing described reagents except API was included in parallels and used as a corrective “blank” at the corresponding time point to ensure that the UV signal of API could be differentiated from the spectral noise of the biorelevant media. Concentration-time profiles were monitored over at least 180 min, in triplicate for each drug unless otherwise mentioned.

The flux (*J*) across the membrane representing the amount (*m*) of material crossing one unit area (*A*) of the membrane per unit time (*t*), was calculated from the concentration-time profiles in the receiver compartments:


(1)

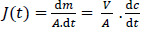



*A* is the area of the membrane (1.54 cm^2^), *V* is the volume of the receiver compartment (20 mL) and d*c*/d*t* (μg mL^-1^ min^-1^) is the slope of the concentration time profile of the drug in the receiver compartment.

The flux values were calculated by fitting the concentration-time profile in the receivers to a straight line to determine the slope and then normalizing the slope to the volume and surface area of the membrane following equation (1). Time intervals to calculate the flux were selected based on the apparent linearity of the concentration-time profile in the receivers, capturing the initial flux process and excluding the lag time unless otherwise is stated.

The solubility in biorelevant media was determined in our lab for such compounds where the solubility could not be found in the literature. Solubility measurements were performed by adding an excess amount of drug into FaSSIF or FeSSIF media in a glass vial. The vials were placed on an end over end rotator into a 37 °C oven for 24 hours. After equilibration, samples were centrifuged/filtered using NANOSEP with 0.2 μm Bio-inert centrifugal devices (PAL Corporation). The filtrate was immediately diluted accordingly into acetonitrile/water 50/50 and analyzed by HPLC.

## Results and discussion

Model drugs categorized as BCS I - IV classes were selected. The drugs included mono- and diprotic acids and bases as well as non-ionizable compounds. UV properties of the samples were also taken into consideration, selecting compounds with sufficient UV chromophores to reduce spectral interaction between DS and strongly absorbing biorelevant media during *in situ* concentration monitoring in donor compartments of μFLUX pairs. The physicochemical properties of the model compounds, p*K*_a_, log *P* [18,19] and solubility in biorelevant media [20-26] were collected from the literature and are depicted in Table 2. The solubility was also determined in our lab for such compounds where the solubility could not be found in the literature.

Table 3 compares the physicochemical properties, the food effect from clinical data and the measured flux of the model compounds. The solubility ratio (fed/fasted) was calculated from the experimental solubility in biorelevant media obtained from the literature (Table 2). The PK parameters, the total drug exposure AUC, and the maximum concentration *C*_max_ in the fasted and fed state were obtained from the clinical data published in the literature and the ratio of geometric means between fed and fasted state was calculated. The food effect was assigned based on the AUC and *C*_max_ ratios. The food effect on the extend of absorption reported in the clinical studies were listed into three categories [12]: positive food effect (AUC and/or *C*_max_ increase with food, 4 compounds), negative food effect (decrease in AUC and/or *C*_max_ with food, 4 compounds) and no food effect (no significant difference with food, 3 compounds). The concentration time profiles obtained in the receiver chambers of μFLUX pairs were used to calculate flux values corresponding to the dissolution in the donor chambers using FaSSIF or FeSSIF media. The flux ratio was calculated as the ratio of flux from the fed (FeSSIF) and fasted (FaSSIF) state. The dose of the marketed product used in the clinical studies and the dose used in the flux measurements are also listed in Table 3.

It is evident from Table 3 that for most of the model compounds, *in vitro* experiment i.e., flux ratio predicted the FE observed in the clinic. The results for each model compound are discussed below.

Amiodarone, anti-arrythmic drug, is a BCS class II drug with ~2.2-fold higher solubility in FeSSIF than in FaSSIF. The flux ratio (FeSSIF/FaSSIF) was determined to be 4.25 at earlier time point interval and 2.21 at later time point interval (Figure S1; supporting information). Both, the solubility ratio, and the flux ratio indicated a positive food effect. The ratio of means of AUC and *C*_max_ between the fed and fasted state was calculated to be 2.36 and 3.68 respectively and confirmed the positive food effect [27]. The drug was reported to be released completely and rapidly from the formulation in the fed state compared to the fasted conditions, thereby reducing the *T*_max_ value from 7.1 to 4.5 hours [27]. An increased bile salt and lecithin production in the fed state is reported to help in solubilization of poorly soluble drugs [28]. Amiodarone has high log *P* value of 7.80 and is believed to undergo micellar solubilized in the fed state [27], that resulted in an increased exposure. On similar lines three other BCS – II drugs, celecoxib [29], danazol [30], and griseofulvin [31] exhibited positive food effect in the clinic and same was predicted by the solubility and the flux ratio between FeSSIF and FaSSIF (Table 3; Figure S2, S3, S4; supporting information).

**Figure 1.**
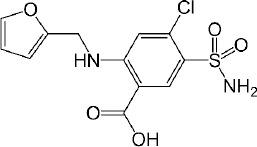
Furosemide

Furosemide is a BCS class IV, diprotic weak acid with p*K*a values at 3.53 (-COOH) and 9.90 (-SO_2_NH_2_) (Figure 1). Since only -COOH group is located within the physiological pH range, the effect of the higher p*K*a (9.90) on ionization and permeability-solubility interplay of furosemide during flux experiments was disregarded for the purposes of this study. The species distribution curve [25] indicates that the molecule is fully protonated at pH 1.2 [AH_2_]^0^ and reaches fully deprotonated state [AH] ^-^ at pH 6.5, while at pH 5.0 only ~ 97 % of the drug is deprotonated. Thus, based on the Henderson–Hasselbalch equation, thermodynamic solubility of the drug is expected to be lower at pH 5.0 than at pH 6.5. The solubility ratio of furosemide between FeSSIF (684 μg/mL) and FaSSIF (3201 μg/mL) was reported to be 0.21 and that agrees with the negative food effect reported in the clinic [32]. It has been reported that the mucus layer present at the surface of the intestinal lining is associated with the lower microclimate pH than that of bulk phase in the intestine. The microclimate pH was reported to be 5.2 – 6.7, when the bulk pH was 7.2 [33]. As per the pH-partition hypothesis, only the uncharged species can permeate through the lipophilic membranes, thus it is expected, that furosemide has higher membrane permeability at pH 5.0 comparing to pH 6.5 where the drug is fully deprotonated. As microclimate pH is expected to be constant (acidic than the bulk pH) in both the fed and the fasting state [33], the extent of furosemide protonation and hence the degree of the absorption flux is anticipated to be same through the lipophilic epithelial membrane, irrespective of the prandial state. This is evident from the clinical data as the AUC ratio (fed/fasted) was reported to be 1.18 (table 3). Interestingly, the negative food effect of furosemide was based on the lower *C*_max_ value in fed state only. The lower *C*_max_ value in fed state might be due to delay in the absorption, possibly due to an interaction/complexation of the drug with bile salts/acids.

**Figure 2.**
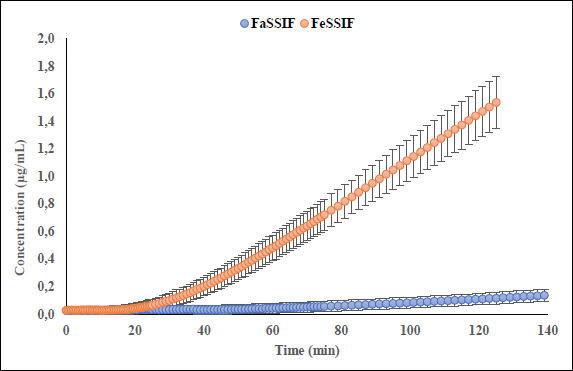
Appearance kinetic of furosemide in the receiver chambers during μFLUX experiment. The corresponding donor chambers of μFLUX pairs were filled with FaSSIF or FeSSIF media. The flux was higher while using FeSSIF medium.

**Figure 3.**
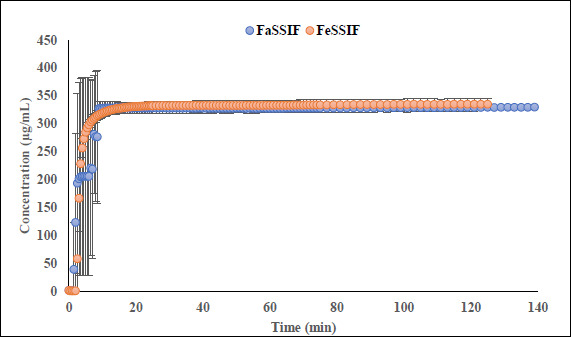
Dissolution kinetic of furosemide in donor compartments of μFLUX pairs.

Interestingly, the flux ratio between FeSSIF and FaSSIF for furosemide was determined to be 11.48 indicating a positive food effect. The food effect prediction using flux ratio was not in agreement with either the solubility ratio or with the clinical results. The solubility in blank FeSSIF (pH 5.0), and blank FaSSIF (pH 6.5) are reported to be 426 and 3017 μg/mL respectively [25]. The bile salts and the lecithin present in the FeSSIF and FaSSIF did not improve the solubility of the drug, possibly due to the electrostatic repulsion between the furosemide ion and the negative charge on the taurocholate/lecithin micelles [25]. Therefore, it is evident that the ionization is playing a major role in the solubilization of furosemide than the bile acid and salts. As the experimental setup could not replicate the *in vivo* microclimate pH as mentioned above, as per the pH-partition hypothesis the flux data obtained during μFLUX experiments showed higher flux in FeSSIF medium (Figure 2) despite of the higher solubility of the drug in FaSSIF, and identical dissolution in both media at the level of drug dose tested (Figure 3). Also, the sink buffer pH on receiver side is 7.4, and as soon as unionized furosemide reaches the receiver compartment, the molecule ionizes, and believed to create an “ion-trap” sink [34]. Therefore, despite of the high solubility of furosemide in FaSSIF, we observed high diffusion flux in FeSSIF medium during *in vitro* experiment. Therefore, it is important to keep in mind the impact of the extent of ionization of the drug, as the pH difference between FaSSIF and FeSSIF may lead to misprediction of the FE.

Isoniazid (INH) is an anti-tubercular BCS-I hydrophilic drug with very high solubility (>60 mg/mL) in both FaSSIF and FeSSIF media. Isoniazid absorption is reported to be reduced following food intake. INH is reported to be converted to a hydrazone species in fed state, making it less available for absorption. Though all types of meals are reported to reduce the isoniazid absorption, the ingestion of a high carbohydrate meal reduced the absorption to maximum extent [35,36]. The formation of the hydrazone species in fed state and hence reduced absorption appears to be the reason for the negative food effect observed in clinical studies [37]. The observed clinical Fed/Fasting AUC and C_max_ ratios were 0.88 and 0.49 respectively. INH has a basic ionizable group with p*K*_a_ 3.8, thus in FeSSIF and FaSSIF media the drug is mainly existing in deprotonated, neutral form. In agreement with pH-partition hypothesis, the diffusion flux ratio between FeSSIF and FaSSIF did not indicate the extent of negative food effect that was observed in the clinic (Figure S5; supporting information). We speculate that the absence/slow hydrazone formation in the biorelevant medium (FeSSIF), might be the reason for not observing the negative food effect during *in vitro* flux measurement. When there is intestinal metabolism or the influx/efflux transporters are involved in the drug absorption, the *in vitro* dissolution/flux might not be able to predict the *in vivo* food effect since the lipid-coated artificial membrane is not designed to mimic the paracellular junction or active membrane transport.

Nefazodone HCl (NZD), an antidepressant, is reported to exhibit negative food effect in clinic [38]. It is a lipophilic diprotic base which has two ionizable groups at p*K*_a_ at 2.1 and 6.7. Since at pH 6.5 the drug is more de-protonated than at pH 5.0, it is expected to observe higher membrane permeability at pH 6.5 comparing to pH 5.0 in aqueous buffers. Nefazodone HCl is a BCS-II drug, showing a low solubility and high permeability. The solubility ratio between FeSSIF and FaSSIF was determined to be 1.52 indicating a positive food effect (Table 3) unlike the negative food effect reported in the clinic. The bile salts, phospholipids, and cholesterol concentration are higher in fed state *in vivo* and in simulated intestine fluid. Bile salts are surface active compounds and when their concentration is above the critical micelle concentration, bile salts aggregate and form micelles. These micelles help in solubilization of drugs with low aqueous solubility [39]. But solubilization of drugs may not always increase their absorption. The bile micelles may “entrap” a drug molecule thereby reducing the concentration of free drug available at the epithelial membrane surface. Micelle formation may also reduce the diffusion coefficient of the drug in the unstirred water layer. The flux experiments were conducted for NZD in two ways, introducing sample in the donor compartments as a dry powder of unformulated material and as a “slurry” of DS in the corresponding biorelevant media at concentration 0.4 mg/mL. The sample, assayed as a dry powder showed poor wettability in FaSSIF comparing to FeSSIF creating “grease balls”, that significantly reduced the surface area of the drug significantly slowing down the dissolution process. However, assayed in form of “slurry”, the flux ratio between FeSSIF and FaSSIF was determined to be 0.74, indicating a negative food effect (Table 3; Figure S6; supporting information). It is imperative to note that the wettability and surface area of a drug substance may influence the results in flux assays conducted for unformulated drug substance compared to a drug formulation. The relationships between the limiting steps of oral absorption (permeability limited, dissolution rate limited, and solubility limited), and the food effects by bile micelles have been outlined and discussed [40].

**Figure 4.**
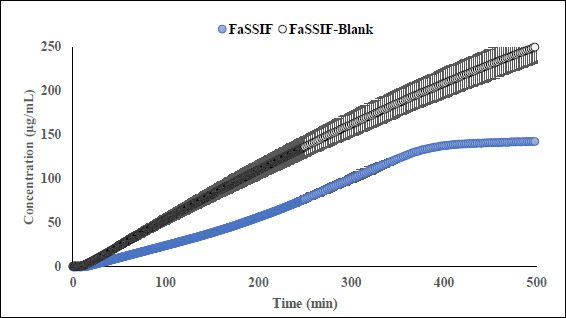
Appearance kinetic of fluoxetine in the receiver chambers during μFLUX experiment at dose equivalent to 200 mg of free base. The corresponding donor chambers of μFLUX pairs were filled with FaSSIF and FaSSIF blank media.

Fluoxetine HCl (FXT) is BCS-I selective serotonin reuptake inhibitor is used to treat depression, obsessive compulsive disorder as well as some other forms of eating disorders. The clinical studies demonstrated that extend of absorption of FXT is not influenced by presence or absence of food reporting fed/fasting AUC and *C*_max_ ratios 0.96 and 0.85 respectively [41]. FXT is a monoprotic highly lipophilic weak base with pK_a_ 9.62. In physiological pH range the compound is mostly remains in its protonated state reaching fully deprotonated state above pH 9.7. Despite high lipophilicity, compound reported to be soluble in FaSSIF (1380 μg/mL) and FeSSIF (1620 μg/mL), showing no food effect based on fed/fasted solubility ratio 1.17 what correlates with fed/fasted AUC ratios reported in clinic. Data collected during flux experiments shows that in the presence of the lipophilic barrier, more complex processes may occur in donor, especially in FaSSIF medium. To better understand an intricate solubility-permeability interplay observed *in vitro*, flux experiments were conducted at several doses of unformulated drug substance (equivalent to 20 mg, 40 mg, and 200 mg dose) in FaSSIF, FeSSIF and in the corresponding plain buffers (herein referred as FaSSIF blank and FeSSIF blank). A representative dissolution profile for 40 mg dose has been depicted in Figure S10; supporting information. Flux values in the FeSSIF blank are higher at all doses comparing to flux in the corresponding biorelevant medium. This suggests that FXT is likely to form micelles with biorelevant` components (lecithin and bile) of FeSSIF. Since only unbound form of compound can permeate through the lipophilic barrier, the higher flux is observed in FeSSIF blank where concentration of unbound fraction is higher in the absence of lecithin and sodium taurocholate. The dissolution and appearance kinetics (in receiver compartment) of FXT in flux experiments conducted at the fasted state depends on dose. While at the dose equivalent 20 mg of free base the appearance rate is linear in FaSSIF and FaSSIF blank, at the dose equivalent 200 mg of free base the flux is significantly reduced in FaSSIF after ~ 5 hours duration of the experiment comparing to FaSSIF blank (Figure 4). The observed reduction of flux in the receivers, correlates with decrease of turbidity and haziness of FaSSIF medium in the corresponding donor compartments of the μFLUX pairs observed after several hours’ duration of the assay. Simultaneously, spectra of FXT in FaSSIF where slowly changing from its original shape making quantification of the sample in donors inaccurate. It is possible to speculate that FXT forms stronger binding with the biorelevant components of the medium at the later stages on experiment comparing to the initial phase.

Fed/Fasted ratio 0.5 calculated based on the flux data in biorelevant media indicates negative food effect at all doses except dose equivalent 200 mg at the late stage of experiments. At 200 mg dose, the fed/fasted ratio increases up to 7 due to suppression of flux in FaSSIF. Fed/Fasted ratio obtained in the corresponding blank media in the absence of lecithin and bile shows ratios 1.0 (no food effect) and 0.5 (negative food effect) at 20 mg and 200 mg dose equivalent correspondingly. Generally, for BCS I drugs, irrespective of the prandial state, the drugs are completely absorbed and hence exhibit no FE. Therefore, the FE prediction based on the μFLUX method may not be reliable for BCS I compounds.

Zidovudine, an anti-HIV drug, falls in BCS-III category. The compound is reported to have a negative food effect [42]. But looking at the PK data, the fed/fasting AUC ratio is 0.9, and that is well within the range of bioequivalence, as defined by FDA. On the other hand, the ratio of *C*_max_ between fed and fasting was 0.3. The flux ratio between fed and fasting state was determined to be 0.92 indicating no food effect (Figure S7; supporting information). The reason for this discrepancy might be due to reported delayed gastric emptying in fed state [42]. The delayed gastric emptying might be responsible for the lower *C*_max_ in the fed state, but almost no change in AUC. Unfortunately, *in vitro* flux measurement cannot predict the change in the physiology due to the presence of food.

For clopidogrel bisulfate [43] and nifedipine [44], the solubility ratio (fed/fasted) was found to be 3.85 and 3.2, respectively. If one goes by the solubility data alone, both the compounds were expected to show a positive food effect. But the clinical data reported no food effect in both the cases. For clopidogrel bisulfate and nifedipine, the flux ratio (fed/fasted) was determined to be 1.24 and 1.01, respectively (Table 3). In both the cases, flux ratio predicted no food effect and agreed with the clinical results (Figure S8 and S9; supporting information). In fed state, the micelles and colloidal species formed by bile salts and lecithin might have helped in solubilization of these two insoluble drugs. The drug molecules may form a complex with bile salts or might get “entrapped” into the micellar core. As only the free drug molecules in solution (unbound form) of the drug is available for the absorption through the epithelial membrane, the total drug solubility may not be a reliable way to predict the food effect. This clearly exhibits that in addition to the drug dissolution in biorelevant media, the evaluation of the diffusion flux where only the unbound drug crosses the membrane, is more reliable way to predict the food effect.

**Figure 5.**
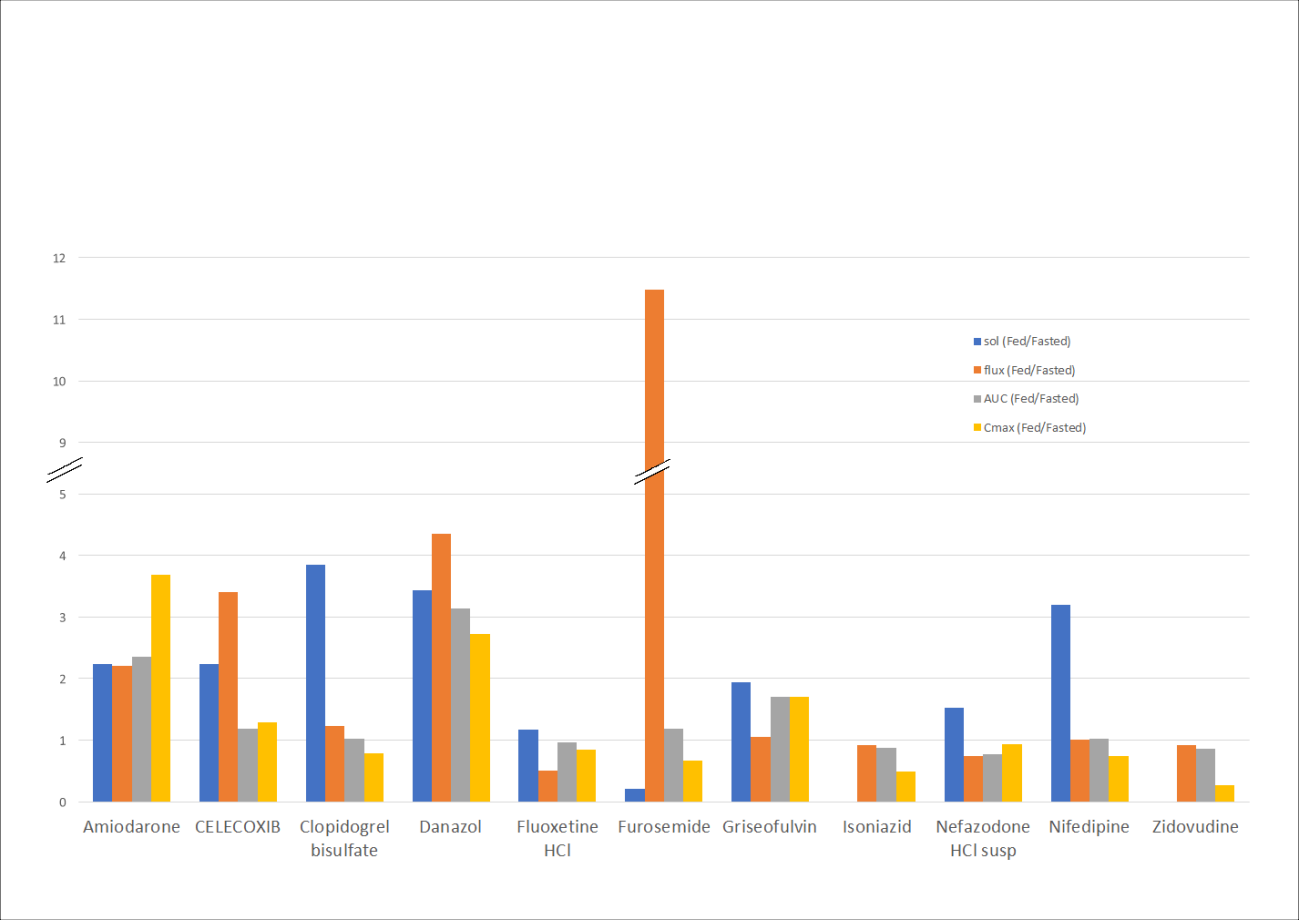
Solubility ratio, flux ratio, AUC ratio and *C*_max_ ratio for the studied compounds

For a side-by-side comparison of the solubility ratio and the flux ratio to the AUC ratio and the *C*_max_ ratio for the studied compounds, a bar graph was drawn (Figure 5). This graph clearly shows that the flux ratio is more predictive of FE than solubility ratio. All compounds that exhibited a positive FE (AUC ratio > 1) in the clinic, also showed a flux ratio > 1. In the case of Amiodarone, the early time interval flux ratio (4.25) was predictive of the initial rate of absorption (*C*_max_ 3.68) while the later time interval flux (2.21) was predictive of the AUC ratio (2.36). For most of the compounds that showed AUC ratio ≤ 1, the flux ratio was also ≤ 1.

During the early stages of drug development, when only a small amount of drug substance (DS) is available, scientists might not be aware of the mechanism of FE and the limiting steps in the oral absorption of a new drug candidate. Also, an increase in drug solubility in FeSSIF medium may not always results in an increase in oral absorption. Using 40 compounds, Kawai et al. [24], demonstrated that the risk of food effect (positive, negative/none) can be assessed by using *in vitro* solubility data and *in vitro* membrane permeability data. The authors used monolayer of Madin–Darby canine kidney (MDCK) II cells for the membrane permeability study. The authors successfully conducted the risk assessment of food effect based on the physicochemical properties of the compounds.

In the present research, we evaluated the diffusion flux (membrane transport rate) across artificial, lipid coated membrane, using side-by-side donor and receiver chambers. The MDCK II monolayer is expensive, and it is time consuming to grow the layer as compared to the artificial lipid coated membrane, for the permeability study. Secondly, one can determine the kinetic solubility (dissolution) and diffusion flux in single setup using side-by-side donor and receiver chambers. Overall, the dissolution/flux set-up appears to be a useful and better tool to predict the food effect than the solubility ratio. As no *in vitro* technique can predict the intestinal drug metabolism (if any), or the impact of the transporters involved in the drug absorption, the dissolution/flux set-up could not predict the negative food effect successfully. Also, it is imperative to note that we compared the published clinical data that was generated using the formulated drug product with that of *in vitro* data generated using unformulated dug substance. This might be the reason for the quantitative differences observed between the *in vitro* data and the published clinical data. But the combination of dissolution/flux (diffusion flux measurement across artificial lipid barrier) may provide very good qualitative insight about the food effect of permeability limited, dissolution rate limited and solubility limited compounds.

## Conclusion

In the present research, using 11 model compounds with diverse physicochemical and pharmacokinetic properties, we demonstrate that a combination of dissolution and permeation (diffusion flux measurement) has better food effect predictive capability than the solubility/dissolution alone. The flux-based method with lipid-coated artificial membrane was found to be more reliable than the solubility/dissolution alone in predicting the food effect in the cases where the drug absorption was driven by passive transcellular diffusion. The combination of dissolution and diffusion flux may not reliably predict the food effect in case of drugs undergoing intestinal metabolism or where transporters are involved in the drug absorption. But the technique provides good qualitative information about the food effect at very early stages of drug development that may help in designing a clinical plan by adjusting the drug dose in fed state.



## Figures and Tables

**Table 1.** Composition of the biorelevant media level I [16]

	FaSSIF	FeSSIF
Sodium taurocholate (mM)	3	15
Lecithin (mM)	0.75	3.75
Monobasic sodium phosphate (mM)	28.36	-
Glacial acetic acid (mM)	-	144.00
Sodium hydroxide (mM)	~13.8	101
Sodium chloride (mM)	106	173
Characteristic		
pH	6.5	5
Osmolality (mOsm kg^-1^)	270 ± 10	635 ± 10
Buffer capacity (mmol L^-1^ DpH^-1^)	12	76 ± 2

**Table 2.** Experimental and predicted p*K*_a_, log *P* and solubility of the study compounds

Model Drug	BCS Class	Type	p*K*_a_	log *P*	Solubility [20-26]	
					FaSSIF (pH 6.5) (μg/mL)	FeSSIF (pH 5.0) (μg/mL)
Amiodarone	II	B	10.24 ± 0.15^a^	7.80^a^	351	784
Celecoxib	II	A	9.38 ± 0.08^a^	3.02^b^	46.2	103.3
Clopidogrel bisulfate	II/IV	B	4.6^b^	4.2^b^	130	500
danazol	II	N	---	4.70 +/- 0.43^b^	8.4	28.8
Fluoxetine hydrochloride	I	B	9.62^a^	4.50^a^	1380	1620
Furosemide	IV	AA	9.90 ± 0.04^a^ 3.53 ± 0.06^a^	2.56^a^	3201	684
Griseofulvin	II	N	---	2.20^a^	23.4	29.2
Isoniazid	I	B	3.8^b^	-0.52^b^	> 60,000	> 60,000
Nefazodone HCl	II	BB	6.7^b^ 2.1^b^	3.5^b^	252	383
Nifedipine	II	N	---	3.17^a^	14.4	46.1
Zidovudine	III	A	9.40 ± 0.01^a^	0.13^a^	>10,000	>10,000

Type: A - acid; AA - diprotic acid; B - base; BB - diprotic base; N - neutral

^**a**^ Ref. [18]

^**b**^ Ref. [19]

**Table 3.** Physicochemical properties, food effect data and measured flux of the studied compounds

Drugs	BCS Class	log *P*	Solubility ratio	Observed AUC Ratio	Observed *C*_max_ Ratio	FE	Dose used in clinical data	Dose used flux study	Flux Fe (±SD; n=3)	Flux Fa (±SD; n=3)	Flux Ratios
			(Fed/Fasted)	(Fed/Fasted)	(Fed/Fasted)		(mg)	(mg)			(Fed/Fasted)
Amiodarone [27] (early)^1^	II	7.8	2.23	2.36	3.68	positive	600	200	0.073 ± 0.001	0.017± 0.003	4.25
Amiodarone (late)^2^	II	7.8	2.23	2.36	3.68	positive	600	200	1.277± 0.109	0.579 ± 0.095	2.21
Celecoxib [29]	II	3.02	2.24	1.19	1.29	positive	200	50	0.632 ± 0.077	0.186 ± 0.021	3.4
Clopidogrel bisulfate [43]	II/IV	4.2	3.85	1.02	0.79	none	75	75	2.168 ± 0.323	1.751 ± 0.540	1.24
Danazol [30]	II	4.7	3.43	3.13	2.73	positive	100	100	0.352 ± 0.063	0.081 ± 0.016	4.35
Fluoxetine HCl [41]	I	4.5	1.17	0.96	0.85	none	40	40	0.85 ± 0.01	1.77 ± 0.15	0.5
Furosemide [32]	IV	2.56	0.23	1.18	0.67	negative	40	80	0.208 ± 0.036	0.018 ± 0.003	11.48
Griseofulvin [31]	II	2.2	1.24	1.7	2.2 (4 h) 1.7 (8 h)	positive	1000	125	0.218 ± 0.009	0.205 ± 0.011	1.06
Isoniazid [37]	I	-0.52	n/a	0.88	0.49	negative	300	300	0.020 ± 0	0.020 ± 0	1.02/0.92
Nefazodone HCl [38]	II	3.5	1.52	0.78	0.93	negative	200	50	1.077 ± 0.084	0.521 ± 0.179	2.07
Nefazodone HCl suspension	II	3.5	1.52	0.78	0.93	negative	200	100	1.923 ± 0.547	2.610 ± 0.618	0.74
Nifedipine [44]	II	3.17	3.2	1.02	0.74	none	10	25	0.235 ± 0.028	0.233 ± 0.073	1.01
Zidovudine [42]	III	0.13	n/a	0.9	0.3	negative	100	200	0.034 ± 0.002	0.037 ± 0.007	0.92

^1^ “early” flux refers to the initial appearance rate in the acceptors. The early flux was calculated using the data in the first 2 hours of the experiment, selecting time interval for each media excluding lag time.

^2^ “late” flux corresponds to the appearance rate during the later time points in the acceptor. The late flux was calculated using the data in the 2.5-9 hours’ time interval, selecting the intervals guided by linearity of the concentration-time profiles in the receptor
