# Supplementary material for: Food effect risk assessment in preformulation stage using material sparing μFLUX methodology
Source: ADMET DMPK. 2022 Oct 7;10(4):299–314. doi: 10.5599/admet.1476 (PMC9793460; doi:10.5599/admet.1476)
Supplement: Supplementary file 1 [file ADMET-10-1476-S1.pdf]

SUPPORTING INFORMATION

Food effect risk assessment in preformulation stage using material sparing µFLUX methodology

Corinne Jankovsky[1], Oksana Tsinman[2], Naveen K. Thakral[1]* #*

1. *Boehringer Ingelheim Pharmaceuticals, Inc., 900 Ridgebury Road, Ridgefield, Connecticut 06877, United States*
2. *Pion Inc., 10 Cook St. Billerica, Massachusetts 01821, USA*

**Corresponding Authors*

#Presently at: Schrodinger, Inc., 1540 Broadway, New York, New York 10036, United States

doi: *https://doi.org/10.5599/admet.1476*

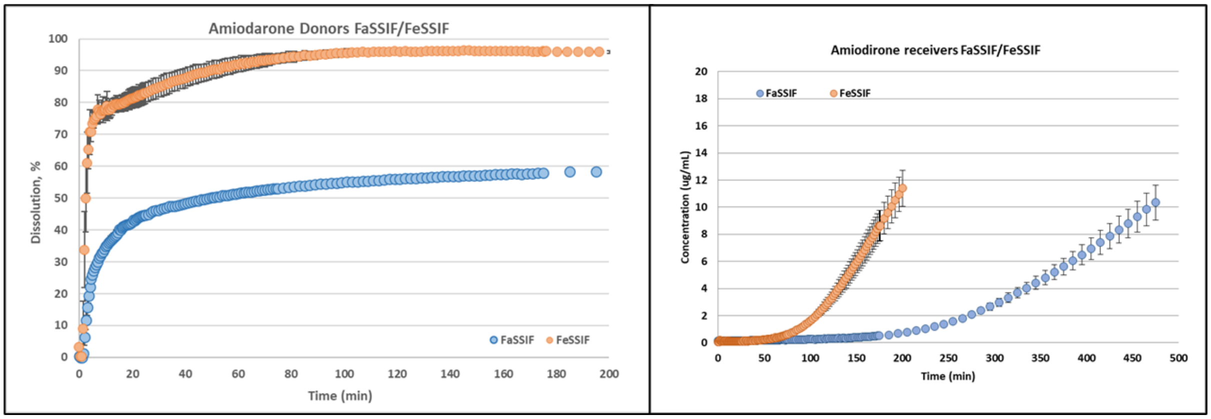

Figure S1: Amiodarone concentration in Donor (dissolution) and Receiver chambers: FaSSIF vs FeSSIF

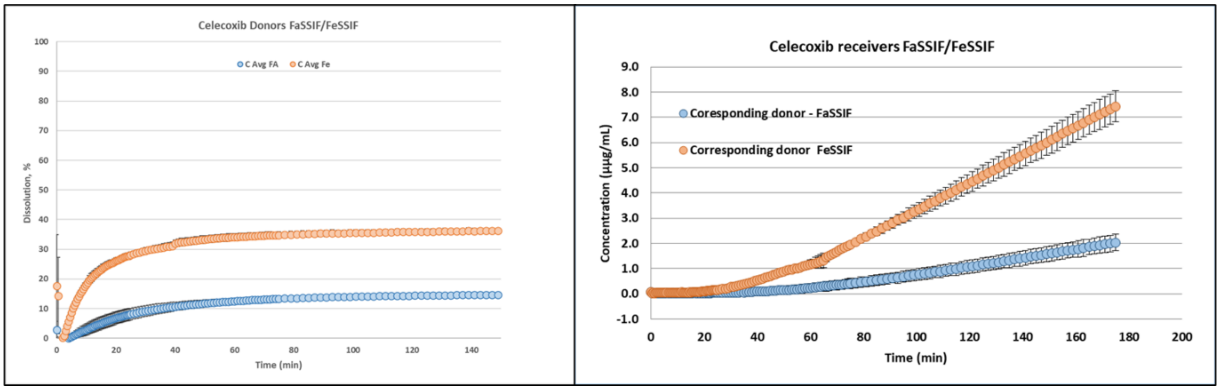

Figure S2: Celecoxib concentration in Donor (dissolution) and Receiver chambers: FaSSIF vs FeSSIF

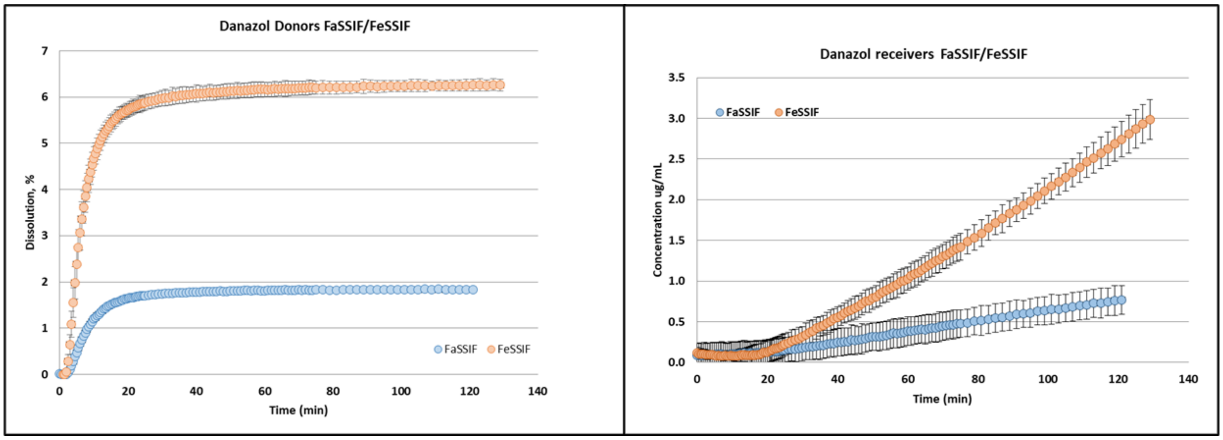

Figure S3: Danazol concentration in Donor (dissolution) and Receiver chambers: FaSSIF vs FeSSIF

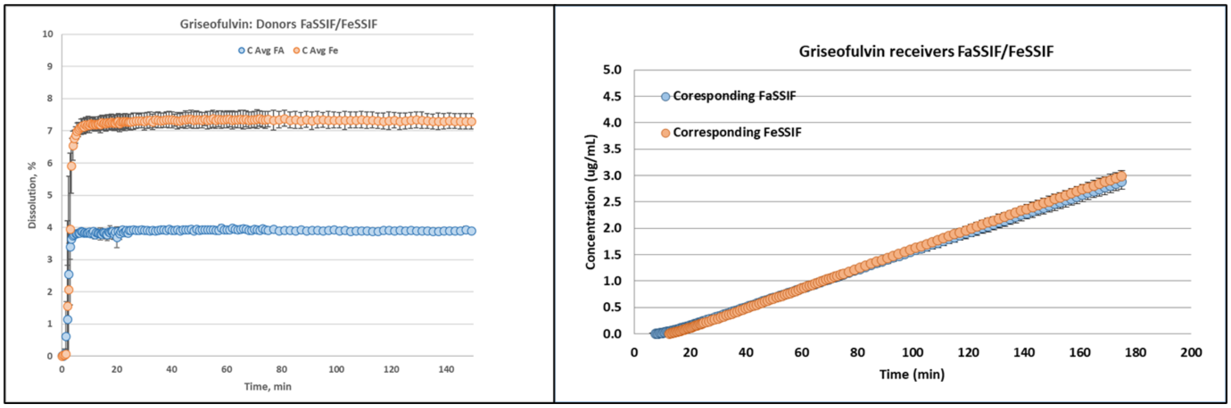

Figure S4: Griseofulvin concentration in Donor (dissolution) and Receiver chambers: FaSSIF vs FeSSIF

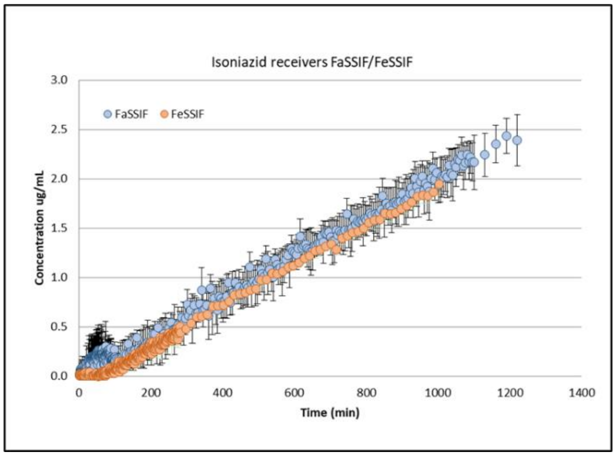

Figure S5: Isoniazid concentration in Receiver chamber: FaSSIF vs FeSSIF. Isoniazid was 100% dissolved in donor chamber within 30 seconds

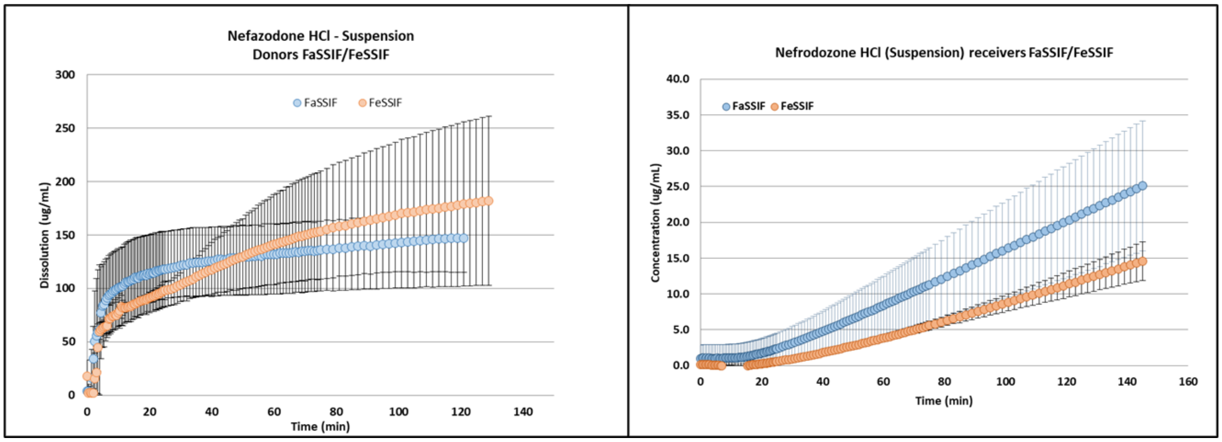

Figure S6: Nefazodone concentration in Donor (dissolution) and Receiver chambers: FaSSIF vs FeSSIF

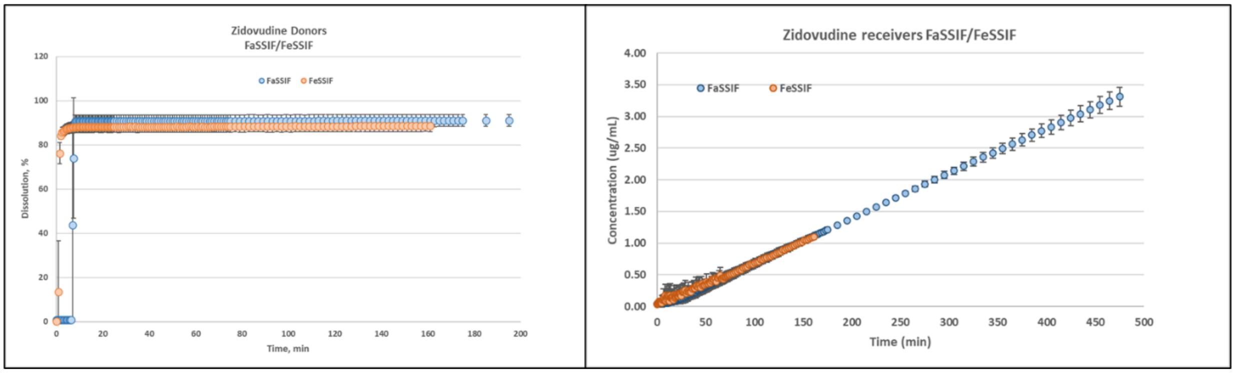

Figure S7: Zidovudine concentration in Donor (dissolution) and Receiver chambers: FaSSIF vs FeSSIF

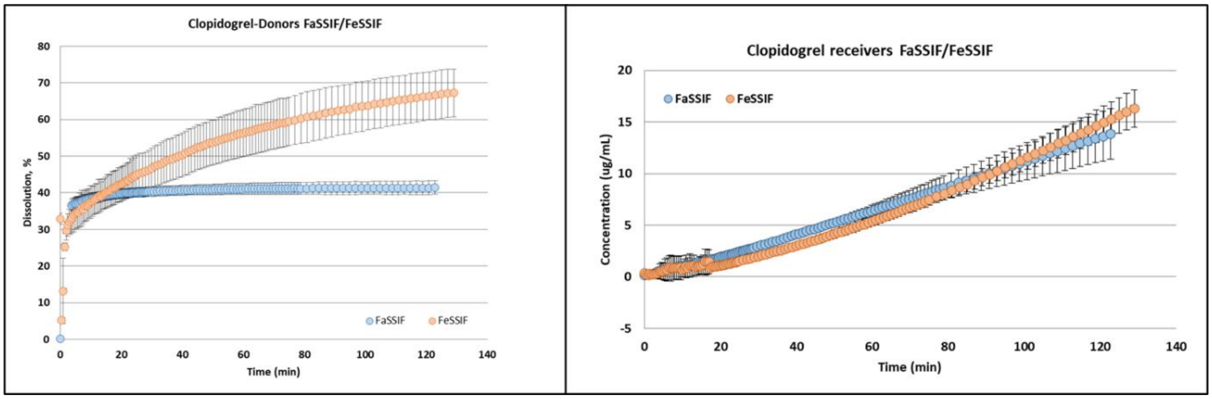

Figure S8: Clopidogrel concentration in Donor (dissolution) and Receiver chambers: FaSSIF vs FeSSIF

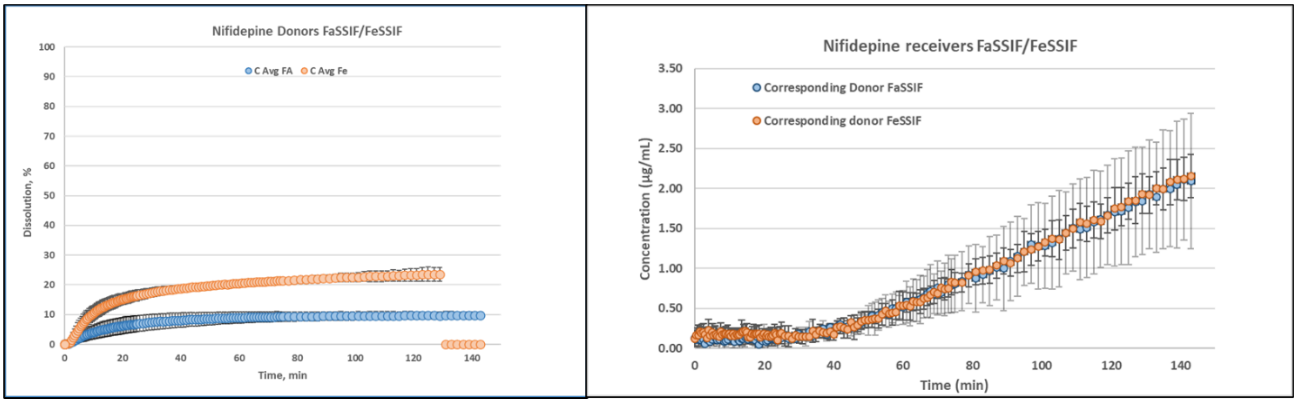

Figure S9: Nifedipine concentration in Donor (dissolution) and Receiver chambers: FaSSIF vs FeSSIF

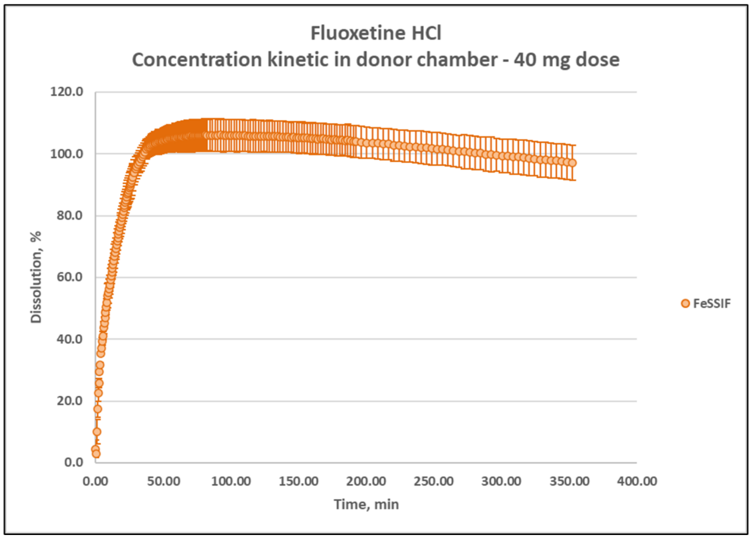

Figure S10: Fluoxetine HCl concentration in Donor (dissolution)
